# Regulator or Driving Force? The Role of Turgor Pressure in Oscillatory Plant Cell Growth

**DOI:** 10.1371/journal.pone.0018549

**Published:** 2011-04-25

**Authors:** Jens H. Kroeger, Rabah Zerzour, Anja Geitmann

**Affiliations:** 1 Department of Physiology, Centre for Nonlinear Dynamics, McGill University, Montréal, Québec, Canada; 2 Département de Sciences Biologiques, Institut de Recherche en Biologie Végétale, Université de Montréal, Montréal, Québec, Canada; Purdue University, United States of America

## Abstract

Turgor generates the stress that leads to the expansion of plant cell walls during cellular growth. This has been formalized by the Lockhart equation, which can be derived from the physical laws of the deformation of viscoelastic materials. However, the experimental evidence for such a direct correlation between growth rate and turgor is inconclusive. This has led to challenges of the Lockhart model. We model the oscillatory growth of pollen tubes to investigate this relationship. We couple the Lockhart equation to the dynamical equations for the change in material properties. We find that the correct implementation of the Lockhart equation within a feedback loop leading to low amplitude oscillatory growth predicts that in this system changes in the global turgor do not influence the average growth rate in a linear manner, consistent with experimental observations. An analytic analysis of our model demonstrates in which regime the average growth rate becomes uncorrelated from the turgor pressure.

## Introduction

**Figure 1:**
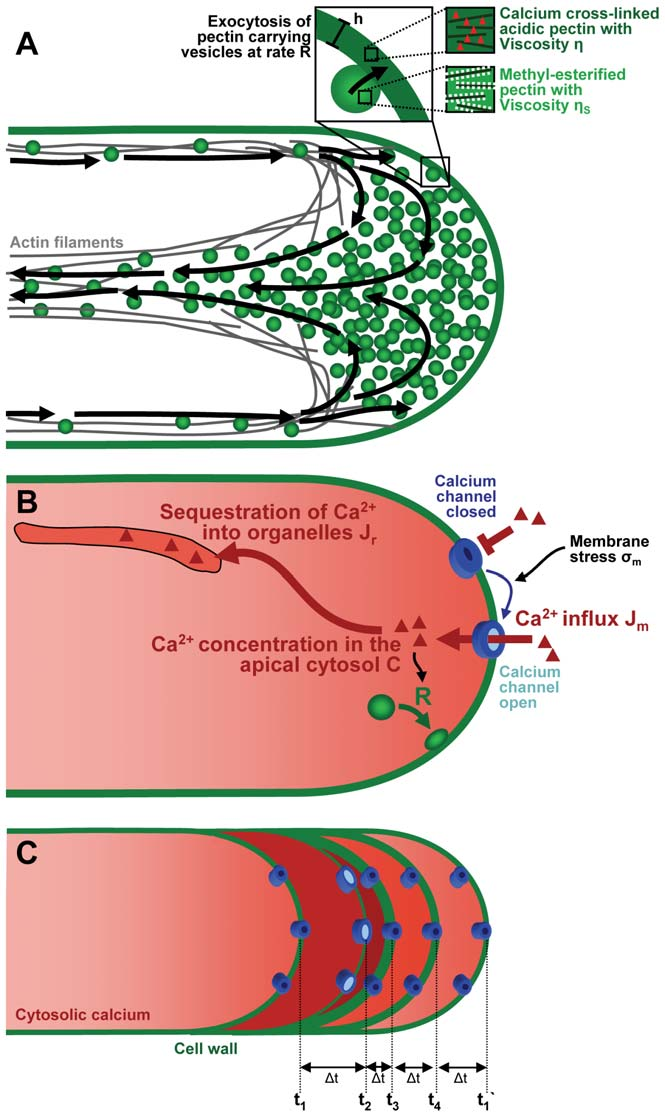
Schematic representation of pollen tube growth. (A) Expansive growth in pollen tubes proceeds by insertion of new cell wall material into the apical cell wall (exocytosis). The new cell wall material (pectins) is delivered to the apical region by actin-mediated transport (black arrows). A continuous and excess flow of vesicles ensures that their availability in the apex is not a limiting factor [59]. The viscosity of the newly deposited wall material is lower than that of the existing wall. (B) Calcium is supplied to the apical cytosol from the outside through plasma membrane located calcium channels. Sequestration into organelles generates a spatial gradient in the cytosolic calcium concentration between the apex and the shank of the tube. (C) Events during one cycle of growth in an oscillating pollen tube. t_1_ through t_4_ indicate four different moments during the cycle ending at t_1_ ` with the time interval Δt being equal between all time points. (t_1_) The tube elongates at an accelerating rate, but the rate of cell wall deformation is below the threshold necessary to open the calcium channels (blue). (t_2_) The cell wall stress and growth rate (elongation rate) are maximal. The calcium channels open, allow calcium influx into the cytoplasm (red) and induce the onset of massive secretion events. (t_3_) Following secretion, the cell wall (green) has reached a high thickness and is under a low stress. The calcium channels are closed. (t_4_) The cell wall stretches as a result of rapid expansion and low secretion. Given that all time intervals Δt are equal, the relative lengths of the arrows are indicative of the different growth rates during the cycle. Objects are not drawn to scale.

The growth of walled cells, such as those composing plants and fungi, is determined by the plastic response of the wall to the mechanical force exerted by the turgor pressure. The force balance between turgor and tensile resistance of the plant cell wall can be affected by manipulating either parameter. The mechanical properties of the cell wall can be modulated by enzymatically altering the degree of cross-linking between existing cell wall polymers or by the addition of new cell wall material. New cell wall material can rigidify the wall, for example through an increase of its thickness or through incorporation of mechanically stable polymers such as cellulose microfibrils or lignin. On the other hand, addition of new cell wall material can also render the wall softer or even liquid, if the added material has low tensile resistance, or if it reduces the overall extensibility of the wall by breaking existing bonds [1]. The addition of softening cell wall material or softening agents is generally carried out through exocytosis, the fusion of carrier vesicles with the plasma membrane. By spatially confining the subcellular location at which exocytosis occurs, the cell manipulates the site of least resistance and hence the location at which cell wall expansion occurs (Fig. 1A) [2]. This is crucial for shape generation in plant cells since the force driving cell wall expansion, internal hydrostatic pressure, is uniform in the entire cytoplasm and acts equally on the entire cellular surface. While many plant cells such as those composing stem or root tissues grow by expanding over their entire surface, others limit growth to small regions. The resulting non-uniform growth events are therefore to a significant degree regulated by the cell's ability to spatially target exocytosis. Tip growing cells such as pollen tubes represent an extreme example of spatially confined growth since cellular expansion is limited to a single very small area at the apex of the growing cell [3], [4], [5], [6]. Micromechanical approaches and mechanical modeling have shown that the pollen tube cell wall is indeed more pliable at the growing end of the cell [7], [8], [9], [10], [11].

The elongation of the pollen tube-a critical process in the sexual reproduction of flowering plants-represents one of the fastest cellular growth processes in the plant kingdom. Its rapid growth generally displays periodical changes in the growth rate [4]. This suggests that the pollen tube does not only exert precise control over the growth process in space but also in time. This temporal control is executed by modulating the force balance between cell wall strength and turgor through feedback loops based on signaling cascades [12]. A feedback loop is a causal path in a signaling network that connects to itself forming a circuit or loop. An initial fluctuation in the value of one component will propagate through the loop until it feeds back unto itself and amplifies or reduces this initial fluctuation. When the amounts of positive feedback (amplification) and negative feedback (suppression) are balanced, stable behaviors such as oscillations in the values of certain components of the system can emerge [13]. Feedback is believed to be involved in the regulation of pollen tube growth as it presents an attractive framework to explain the oscillations observed in the tube growth rate or cellular features such as the apical cytosolic calcium concentration [4]. Particular classes of feedback loops present distinctive behaviors such as the doubling of the oscillation period upon slight parameter changes. This was observed in tobacco pollen tubes [14], [15]. However, other changes in frequency resulting from manipulation of the pollen tube growth environment occur gradually [16], [17], [18], [19], [20], [21].

While many of the variables governing pollen tube growth have been identified, their precise spatio-temporal interaction and the feedback loops that ensure the stability of this far-from-equilibrium and dynamical process remain elusive. In particular, the role of the turgor pressure is a matter of intense debate [14], [15], [22]. While turgor is generally accepted to be the generator of the mechanical force that drives tube elongation [2], the overall pollen tube growth rate does not appear to be directly proportional to the global turgor pressure [23]. This is consistent with other plant cell systems [24]. Furthermore, although periodic increases in the turgor pressure have been postulated to precede phases of rapid growth rate [14], [15], both pressure probe and micro-indentation measurements clearly demonstrated that oscillatory growth in pollen tubes is not accompanied by measurable variations in the turgor [21], [23]. This absence of correlation has led to fundamental questions about the relationship between turgor and pollen tube expansion. These are further inspired by the observation that other tip growing cells such as water molds are able to grow without measurable turgor [25].

Here we investigate the temporal relationship between growth rate and turgor pressure in oscillatory pollen tube growth. We use a mathematical model combining the biomechanics of the tube elongation and the dynamics of the biochemical reactions leading to material delivery and changes in the cell wall. Furthermore, in order to shed light on the importance of turgor in the regulation of the pollen tube growth rate, we experimentally determined how sensitive the growth rate is to changes in the osmotic pressure of the surrounding medium. The predictions made by our model are consistent with experimental findings and provide an explanation for several phenomena that have been at the center of considerable controversy [14], [22], [26].

## Results and Discussion

### Theory and modeling strategy

Plant cell growth has first been represented mathematically by Lockhart [27] who described the relationship between growth rate and turgor pressure. This formalism has been used widely to model the expansion of plant cell walls in response to turgor pressure [3], [28], [29], [30]. In its original form, the Lockhart equation states that the rate of cell wall expansion 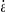 is proportional to the difference between the actual pressure P and a critical threshold value for the pressure Y if P>Y
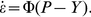
(1)


In the absence of a yield value Y, this relation is identical to the Stokes law of viscous flow [31]. By describing the plant cell wall as a thin hemispherical shell of uniform viscoelastic material, the equation allows us to predict its response to the internal pressure and the resulting stress in the shell. Viscoelastic theory predicts that for low pressures, and thus low stresses, the shell will behave elastically, i.e. it will respond with a finite expansion and return to its initial configuration if the stress is removed. This would correspond to a plant cell that expands by a finite amount, but does not expand continuously. Cells composing pulvini and stomata repeatedly undergo such elastic and reversible deformation during their life time. For a high pressure greater than a critical value, the cell wall material will behave plastically. It will undergo an irreversible expansion with a rate proportional to the pressure P and the extensibility Φ. For the purpose of our model, we define the extensibility as the inverse of the material's viscosity [32]. It is hence a material property that is independent of the cell wall geometry or thickness.

The Lockhart model is supported by observations that decreasing the turgor below a critical level stops pollen tube growth [21], [23] and modulating the osmolarity induces instantaneous and transient variations in the growth rate [33]. It is also in agreement with changes in the instantaneous growth rate in response to transient changes to the cell wall's rheological properties [21]. We therefore base our model on the Lockhart equation and use it to replace Darcy's law that had been at the base of an earlier model of pollen tube growth [34]. The Lockhart equation allows us to account for the cell wall stress, which is not possible using Darcy's law. Furthermore, the present model allows for the change in cell wall viscosity which was not taken into account in this previous model. Other components of the earlier model are maintained, such as the feedback loop coupling growth rate and vesicle secretion.

### Viscoelastic model of cell wall expansion

The Lockhart equation relates the strain rate 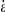 to the cell wall extensibility Φ, the cell wall stress σ and the yield stress σ_y_ σ_y_ through
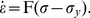
(2)


By modeling the apex of the pollen tube as a thin shell of viscous material [28], the cell wall stress σ can be related to the turgor pressure P, the radius of curvature of the tube r and the cell wall thickness h by
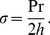
(3)


If the radius of curvature and the cell wall thickness remain constant, then the stress is equivalent to the turgor pressure, and eq. 1 is recovered. Furthermore, the strain rate is related to the tube growth rate v(t) by the relation
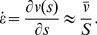
(4)


Here s is the curvilinear coordinate describing the position on the arc starting at the tube's pole and extending rearwards along the shank; and S is the length of this arc. While v(s) is the velocity at each point on the cell wall, 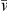 is the average growth rate of the pollen tube which we will designate by v(t) for the remainder of the article. Eq. 1 provides a direct relation between the expansion rate 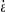 and the turgor P. As discussed in the introduction, the experimental evidence for this direct relationship is inconclusive. The Lockhart equation implies that the growth rate and the turgor are directly correlated if the extensibility 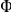 and cell wall thickness h are constant. However, the process of cell wall elongation directly affects the cell wall thickness and indirectly affects other variables such as the cell wall extensibility. Furthermore, cell growth increases the volume of the cytoplasm. Since the pressure is tightly related to the volume of the cytoplasmic fluid, the growing pollen cell must constantly pump water to maintain the turgor pressure. While the temporal change in the turgor remains a debated issue, we assume, in the section *Global turgor changes*, that it is rapidly controlled and thus maintained at a constant value [22], [23].

### Variation in cell wall thickness

The elongation of the pollen tube leads to the stretching and thinning of the cell wall in the apical region of the tube. The elongation is dependent on the Poisson's ratio of the cell wall in the elastic regime. Unless this thinning is balanced by the secretion or deposition of new cell wall material, this process eventually leads to mechanical failure and thus the bursting of the growing pollen tube. The result of concomitant stretching and secretion will be reflected in the dynamics of the cell wall thickness h(t). The rate of change of the cell wall thickness is balanced by an increase through vesicle deposition R and a decrease due to the stretching resulting from growth [34]

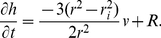
(5)


Here r_i_ denotes the inner radius of the tube apex. The addition of cell wall material through vesicle secretion is assumed to be proportional to the cytosolic calcium concentration C inside the pollen tube apex [35], [36]

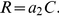
(6)


Thus, in order to properly account for the change in the cell wall thickness, we must estimate the cytosolic calcium concentration at the tip of the pollen tube, and how it varies as a function of time. The parameter a_2_ = 4.1×10^−4^ µm/s/µM is chosen such that a fusion rate of R = 4.1×10^−4^ µm/s [34] is achieved in the presence of an average cytoplasmic calcium concentration of C = 1 µM.

### Calcium dynamics

The cytosolic calcium concentration inside the pollen apex is increased by an influx of calcium ions through the plasma membrane and decreased by the binding of calcium ions with various components of the cytoplasm. These reactions include the sequestration of calcium ions by the endoplasmic reticulum, vacuoles and mitochondria. The calcium dynamics in the pollen tube have previously been modeled by a reaction diffusion equation [34]. Since the calcium channels in the membrane are stretch-activated [37], the influx J_m_ is modulated by the stress on the membrane. The maximal conductance of the channels was evaluated at 15 pS [37]. The rate of change of the calcium concentration is modeled differently at the membrane (x = x_m_) and in the tube far from the membrane (x<x_m_). In the immediate vicinity of the membrane, the rate of change of the cytosolic calcium concentration is proportional to the influx J_m_ [38].
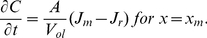
(7)


Here A is the surface area of the membrane covering the tube apex and V_ol_ is the volume of the apex. The term J_m_ denotes ion influx through the channels
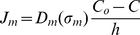
(8)


while the second term 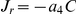 denotes the capture of calcium ions by available sites inside the apical and subapical cytoplasm [34]. The dependence of the stretch-activated channel conductance and thus of the calcium diffusion constant on the membrane stress σ_m_ [39] is given by a sigmoid function
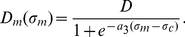
(9)


In the cytosol further away from the membrane, the calcium obeys a standard diffusion-reaction equation [34]

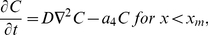
(10)


where the diffusion constant D is assumed constant in time and space. The difference between eqs. 9 and 10 reflects the different geometries of the membrane-located ion channels and the cytoplasm filling the tube apex. Since the ion channels have a much smaller diameter than the pollen tube lumen, the ion movement in the channel will be convective, whereas it is diffusive in the pollen tube cytosol. The ratio of convective to diffusive motion is given by the Peclet number [31].

In the absence of detailed knowledge on the calcium sink in the apex, we assume that the number of available sites is constant in time, such that the binding reaction depends only on the cytosolic calcium concentration. The total electrical conductance of the apex was evaluated at 150 pS [12], [34]. The factor of 10 between the total conductance of the apex and the conductance of individual calcium channels suggests that the number of channels on the tube apex is very limited. Eq. 9 raises the interesting question of the relationship between the stress in the membrane and the stress in the cell wall. The value of the cell wall stress σ can be estimated from eq. 3 [28], [32] and the turgor pressure. For a pressure of P = 0.2 MPa, r = 6 µm and h = 0.1 µm, the stress in the cell wall is of the order of 6 MPa. The maximal stress on the plasma membranes can be estimated from the value of the surface tension γ that will induce rupture (5.7–13.2 J/cm2 [40]). This membrane surface tension γ can be converted into a stress σ_m_ by σ_m_ = γ/2h_m_ [29] which yields a membrane stress of 0.57–1.32 MPa for a membrane thickness h_m_ of 5 nm. It appears that the stress in the membrane must be significantly smaller than the stress in the cell wall. We thus assume that the stress on the membrane is set by the joint movement of the plasma membrane and the cell wall. Due to a no-slip condition at the membrane-cell wall interface, the lipids forming the membrane move at the same velocity as the pectin molecules forming the cell wall. The stress in the membrane is set by the velocity of the lipids and the strain rate of the membrane. The tension in the membrane must obey
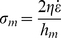
(11)


where η−1×10^−9^ Pa×s×m [41] is the dynamic surface viscosity of the membrane and 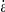 is the strain rate common to the cell wall and the membrane. The use of the surface viscosity renders eq. 11 homogeneous. For a strain rate of 0.1 s^−1^ [32], the stress in the membrane is on the order of 0.4 Pa and thus well below the cell wall stress which ranges in MPa.

### Extensibility and viscosity

When soft cell wall material is secreted, it is incorporated into the existing cell wall. We therefore assume that the cell wall extensibility, i.e. the inverse of the viscosity, changes as a function the secretion rate R [42]. The rate of change of the extensibility is modeled as a mixing process. This modeling strategy is a simplification of the dynamics in the cell wall that include binding to existing polymers and transport driven by turgor pressure [43]. After a series of secretion events, the cell wall viscosity is assumed to be the average of the original highly viscous portion and the added softer (less viscous) portion. In order to calculate the average, the viscosity of the original portion η(t) and the viscosity of the newly added material η_s_ must be weighted by the values of the thickness prior to the secretion h(t) and the added thickness RΔt. However, this process will continuously reduce the viscosity, without accounting for enzyme mediated hardening that occurs during cell wall maturation. A crucial maturation process in the pollen tube cell wall is the de-esterification of pectins by the enzyme pectin methyl esterase that is initiated after the deposition of the polysaccharide at the cellular surface [5], [8], [10]. The removal of methyl-groups leaves negatively charged carboxyl groups, that in the presence of calcium ions, leads to the gelation of the polymers [44]. In order to account for the resulting increase in viscosity, the numerical scheme is supplemented with a linear reaction rate term η_eq_−η(t) with a reaction rate constant k∼1 (2nd term in eqn. 12). Here ηeq is the maximum value for viscosity that can be reached through de-esterification, i.e. when all methyl groups have been removed from the pectin monomers.
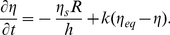
(12)


All simulations were carried out using the simple Euler algorithm. Eqs. 2–9 were coupled by solving them simultaneously with Matlab (The Mathworks). Since we assume that the membrane stress is set by the cell wall motion described by *ν*, the membrane stress σ_m_ in eq. 9 was replaced by the growth rate using eqs. 11 and 4. Accordingly, the critical stress σ_c_ (eq. 9) leading to massive exocytosis is written in terms of a critical growth rate *v_c_*.

### Global turgor changes

**Figure 2:**
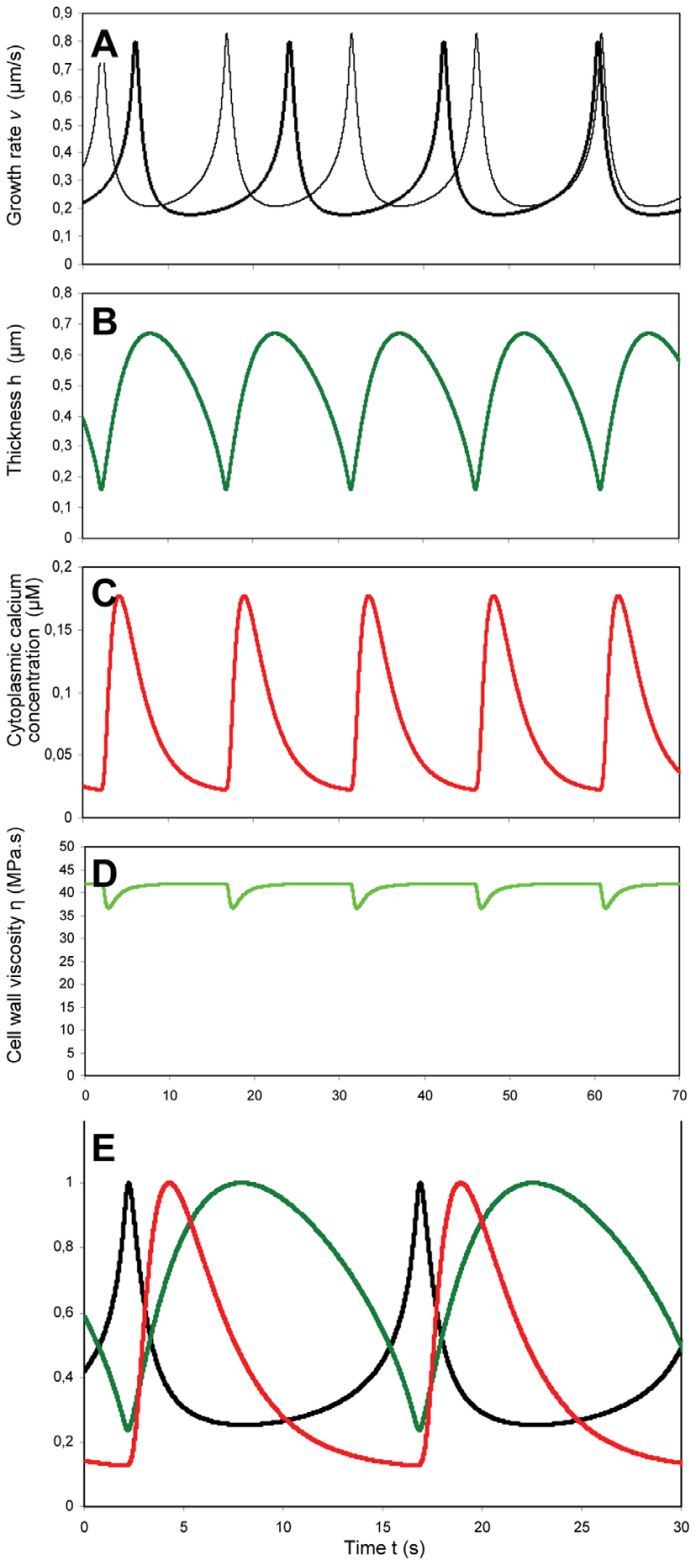
Oscillations predicted by the simulations. (A) Oscillation frequency and amplitude in the pollen tube growth rate depend on the extracellular calcium concentration C_o_ (C_o_ = 1 µM, thick line; C_o_ = 1.5 µM, thin line) (B) Cell wall thickness. (C) Cytosolic calcium concentration in the apex. (D) Cell wall viscosity. (E) Phase shifts are visualized by superimposing normalized growth rate (black), cytosolic calcium concentration (red) and cell wall thickness (green). C_o_ = 1 µM for B-E.

We model the growth of pollen tubes by coupling the Lockhart equation for the growth rate to the dynamical equations for the cytosolic calcium concentration and the secretion rate which feeds back onto the cell wall rheology, thus controlling the growth rate (theory section; Fig. 1B). The identification of material delivery (secretion) and hydrodynamics (Lockhart equation) as the two primary processes controlling tube growth was proposed by Geitmann [45]. We find that the growth rate oscillates with a peak that precedes that of the cytosolic calcium concentration by 2 s (Fig. 2). Given the period length of an oscillatory cycle of 14 s, this phase shift is equivalent to +51° (with positive numbers indicating a delay of a cellular event behind a peak in growth rate). This is consistent with the magnitude and the order of events measured in oscillating *Lilium longiflorum* pollen tubes [18].

**Figure 3:**
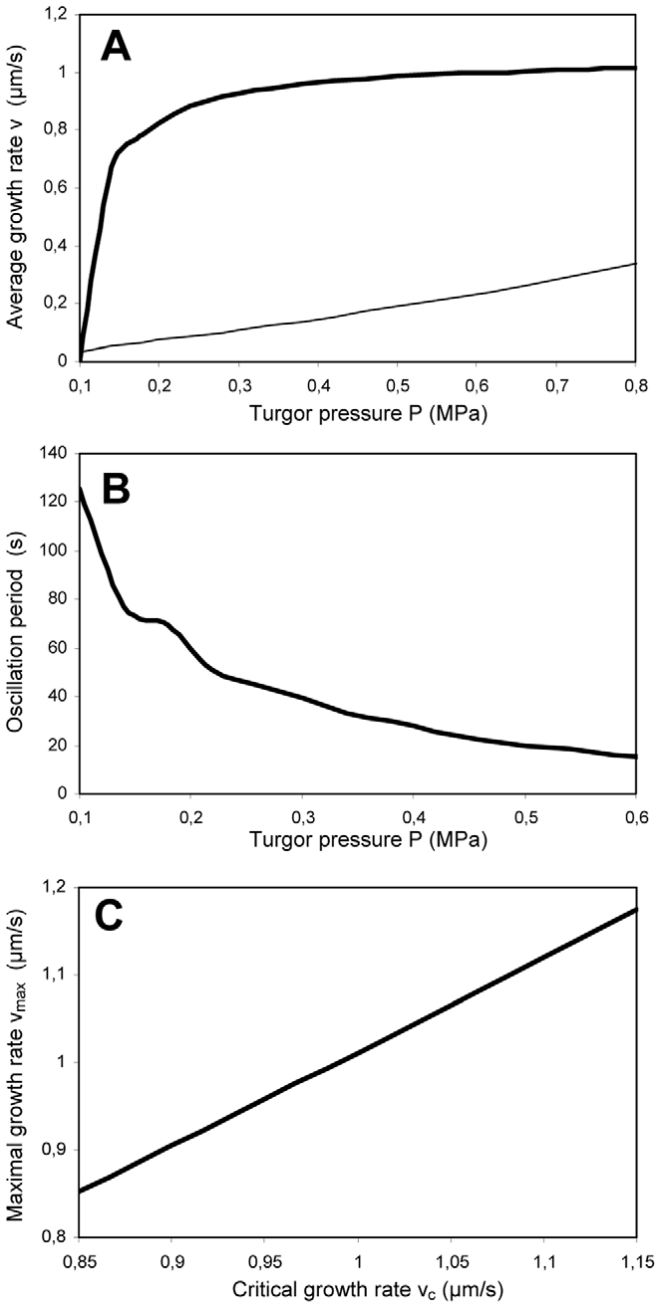
Change in the average growth rate and oscillation period for different turgor values. (A) Variation of average tube growth rate with changing turgor pressure (C_o_ = 0.3 µM, thick line; C_o_ = 12 µM, thin line). (B) Variation of oscillation period with changing turgor pressure (graph shows simulations for C_o_ = 12 µM; periods for C_o_ = 0.3 µM are not shown since they are below 10 s). (C) Maximal growth rate as a function of the critical growth rate at which the calcium channels open.

To study the effect of global turgor changes on the average growth rate, simulations were carried out with different values of the turgor pressure. For small values of the extracellular calcium concentration (C_o_ = 0.3 µM), the growth rate increases as a function of pressure until it reaches a plateau at 0.3 MPa (Fig 3A, thick line). Above 0.3 MPa, the turgor value has a minimal effect on the average growth rate. For turgor values greater than 0.3 MPa, the oscillations in the growth rate have a small amplitude with an average value close to the critical value *v*_c,_ the value of the growth rate at which massive exocytosis resets the cell wall. No matter how high the turgor pressure, the average growth rate cannot increase significantly beyond *v*_c_ and is thus only weakly dependent on the turgor. However, when the oscillation amplitude is large (C_o_ = 12 µM), the average growth rate is proportional to the turgor pressure within the biologically relevant pressure range (Fig. 3A, thin line). This difference in behavior can be explained by a mathematical analysis of our threshold model. It shows how the upper bound on the instantaneous growth rate set by our threshold mechanism prevents the average growth rate from increasing despite an increase in the turgor. The theoretical relationship between the turgor and the average growth rate 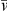 is obtained by a slow-fast analysis [46] of our model (see Text S1) and yields 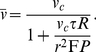
(13)


Here τ is the time necessary for the cell wall to recover its initial state after massive exocytosis events (see Text S1). It appears that the dependence of the average pollen tube growth rate on the turgor pressure P depends on the ratio 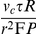. If this ratio is much smaller than 1, the average growth rate will be close to *v*_c_ and essentially independent of P. However, if the ratio is much greater than 1, the average growth rate will be directly proportional to P, i.e.
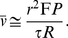
(14)


**Figure 4:**
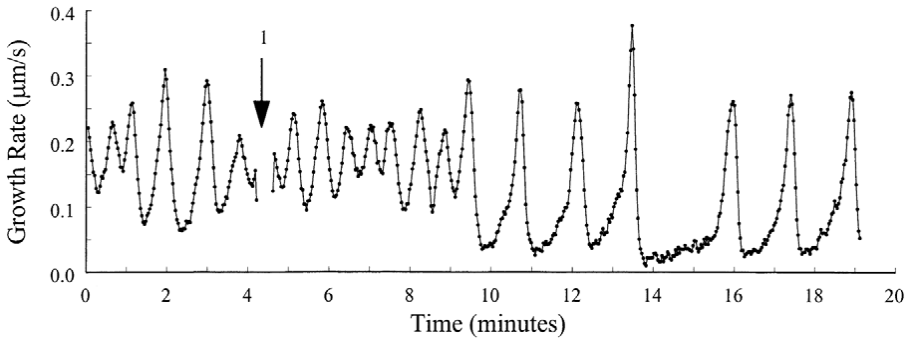
Increase of oscillation amplitude is accompanied by an increase in oscillation period. Raising the concentration of MES, an H^+^ buffer, resulted in a reversible increase in the growth oscillation amplitude and duration while decreasing the frequency in *Lilium longiflorum* pollen tubes several minutes after administration (marked by the arrow). Figure reprinted from [20] with kind permission from the authors and from Springer Science +Business Media.

Reducing the secretion rate R results in a decrease of the amplitude of the oscillations. This behavior is due to the threshold dynamics of our model, i.e. the fact that the cell wall characteristics (thickness and viscosity/extensibility) are reset once their value reaches a threshold value. The average growth rate cannot increase beyond some critical value set by the exocytosis mechanism despite a drastic increase in the turgor pressure. For small growth oscillations, the growth rate will always be close to its maximal (and threshold) value, despite increases in the turgor: the average growth rate will thus be insensitive to the turgor value. These dynamics can be illustrated by a ball bouncing on the ground. In this case, the threshold for the vertical position of the ball is the ground. The bigger the bounces, the longer they last, and the further the average vertical position is from the ground. Conversely, a ball with small bounces will have an average vertical position very close to the ground. A robust prediction of threshold dynamics is that when the oscillation is amplified, the oscillation period increases, and the average value is moved further from the threshold value (corresponding to a decrease in the case of the average growth rate). In our simulations, a decrease in the period does accompany the increase in average growth rate as the turgor pressure is increased (Fig. 3B). This exact behavior was observed upon buffering the pH in the cell wall in *Lilium longiflorum* pollen tubes, which caused the oscillations to increase both in duration and amplitude [20]. This was accompanied by a decrease in the average growth rate by about 30% (Fig. 4).

It is inherent to this threshold model that the maximum growth rate of the pollen tube cannot exceed by much the growth rate that induces the opening of the calcium channels (Fig. 3C). Once the growth rate reaches the value that induces the opening of the calcium channels, a sequence of events is triggered that reduces the growth rate to its minimum value. As will be seen in greater detail in the following section, the minimal value of the growth rate depends on the amount of calcium ions entering the cytosol when the calcium channels open.

### Experimental measure of the average growth rate

**Figure 5:**
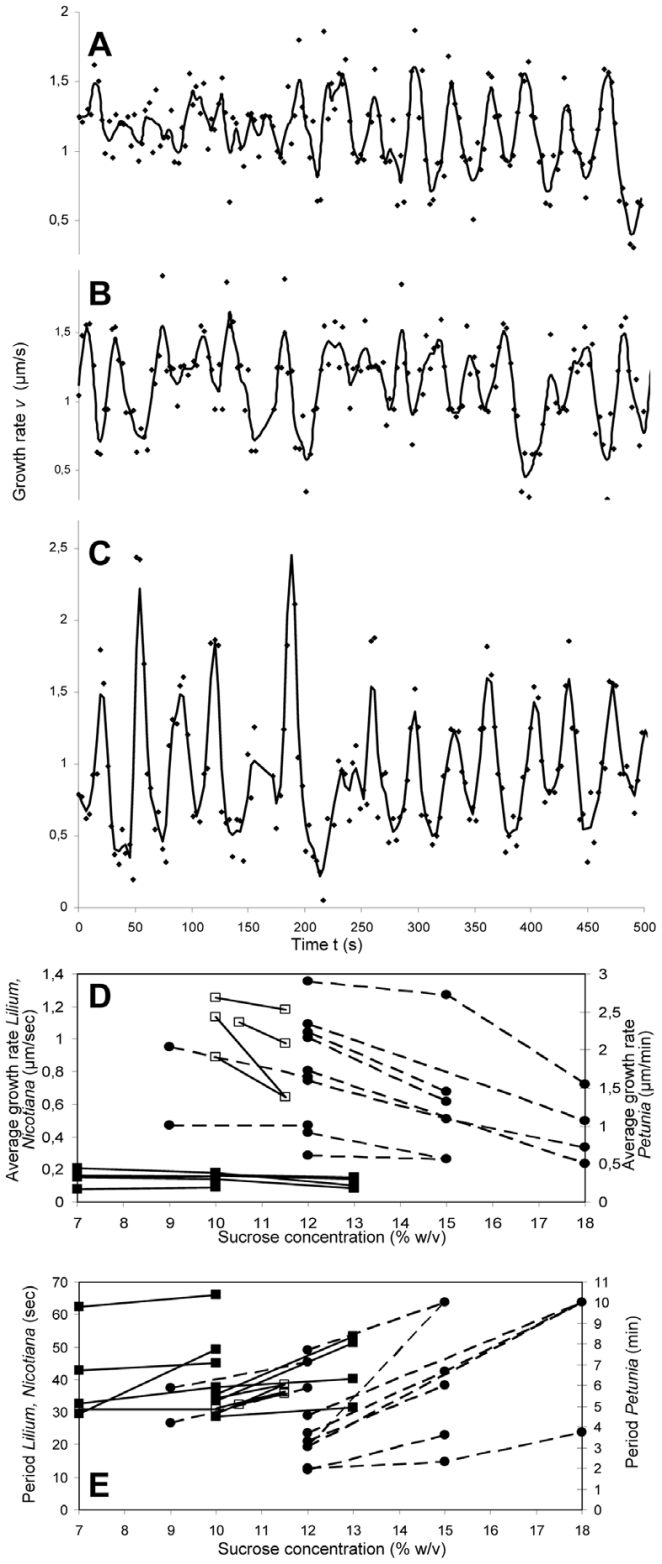
Effect of changing turgor on growth oscillations. Experimental measure of the instantaneous pollen tube growth rate in *Nicotiana tabacum* in a medium with 10 mM sucrose (A), 10.5 mM sucrose (B), and 11.5 mM sucrose (C). (D,E) Average tube growth rate (D) and period of growth oscillations (E) of individual pollen tubes from *Lilium longiflorum* (▪), *Nicotiana tabacum* (□) and *Petunia hybrida* (•) before and after changing the osmotic value of the medium by manipulating the sucrose concentration (*Nicotiana, Petunia*) or by adding mannitol (*Lilium*). For mannitol, the data are plotted against the corresponding sucrose concentrations of equimolar solutions. The graphs compile new data with re-analysed data of experiments performed for [21].

In order to test the predictions of our model, we used *in vitro* growing lily, tobacco and petunia pollen tubes to measure the average growth rate and the period of the growth rate oscillation as a function of the osmotic value of the growth medium. Previous studies had shown that increasing the external osmotic pressure induces a decrease in the cytosolic turgor pressure in pollen tubes [23]. We thus altered the growth medium by modifying either sucrose or mannitol concentrations and recorded the growth rate of germinated pollen tubes (Fig. 5A–C). We observed that an increase in the osmotic value of the medium modestly reduced the average growth rate and modestly increased the period of oscillations of individual tubes (Fig. 5D). The changes in the average growth rate and period of oscillation are statistically significant, as assessed using a one-sided paired t-test (p<0.025). These results are in agreement with previous studies of growth under conditions of changing osmotic values [15]. An increase in growth rate upon a turgor pressure increase is predicted by our model as embodied by equation 14 (see Text S1 for the derivation). Accordingly, the size of the increase in growth rate and decrease in period depend, among others, on the turgor pressure and the amplitude of the growth rate oscillations. When the turgor is only slightly higher than the yield pressure, the model predicts a linear relation between turgor pressure and average growth rate. On the other hand, for high turgor pressures, the model predicts that the average growth rate will become asymptotically independent of turgor as the turgor increases and the growth rate oscillation amplitude decreases. Such an absence of direct correlation between the average growth rate and turgor was reported in *Lilium longiflorum* [23].

### Effect of transient turgor changes

**Figure 6:**
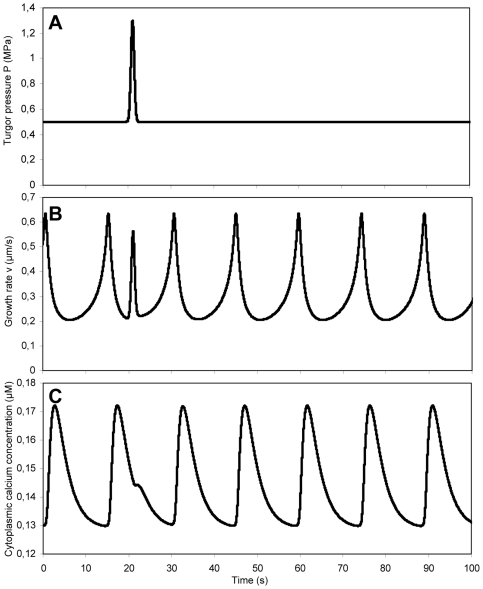
Simulated response of an oscillating pollen tube to a transient perturbation of the turgor pressure. (A) Transient increase in turgor pressure at t = 21 s. (B) Tube growth rate. (C) Apical cytoplasmic calcium concentration.

In order to gain a better understanding of the pollen tube oscillator [12], [16], we simulated transient perturbations of the turgor pressure. We showed above that the average and maximal values of the growth rate are not very sensitive to global, long lasting changes in the turgor pressure. Is this also true for transient (short) changes of the turgor pressure? One can predict that if the pressure transient is of sufficient amplitude and with a timescale much shorter than that of the other quantities, the growth rate should correlate with the pressure during the transient. We simulate a transient increase in the turgor pressure by rendering the turgor time dependent (Fig. 6). The turgor is maintained at 0.5 MPa until, at t = 21 s, it is raised to 1.3 MPa for a very short time (<2 s) (Fig. 6A). This is modeled by the following expression for the turgor pressure given in MPa 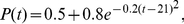
(15)


The cell wall expansion rate upon a transient stress perturbation cannot be modeled using eqs. 1 and 2 which are valid for constant values of the stress. For changing values of the pressure, and thus the stress exerted on the cell wall, the strain rate obeys the augmented growth equation that had been established based on the Lockhart equation [30]

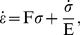
(16)


where 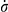 is the rate of change of the cell wall stress and E is Young's modulus. Under conditions of slowly changing cell wall stress such as those modeled in the previous section, the augmented growth equation reduces to the Lockhart equation. Using eq. 12 instead of eq. 2, we observe an instantaneous increase in the growth rate that is proportional to the increase in turgor (Fig. 6B), as prescribed by the Lockhart equation. The perturbation of the turgor pressure is accompanied by a sudden influx of calcium ions (Fig. 6C). We conclude that contrary to the behavior of the average growth rate upon permanent changes in turgor, for very short and transient turgor variations the instantaneous value of the growth is directly proportional to the turgor. Crucially, this confirms that the cell wall expansion in pollen tubes obeys the Lockhart (i.e. the augmented growth) equation despite the lack of correlation between the average growth rate and the turgor.

These simulations help us to understand the lack of sensitivity of the average tube growth rate to global changes in the turgor. Our model suggests that the maximal value of the tube growth rate depends on the membrane strain rate at which the calcium channels open (Fig. 3C). Specifically, the cell wall expansion rate cannot increase much beyond the strain rate that will open the channels. If turgor is very high and the oscillations in the growth rate have a small amplitude, the average growth rate will be close to the threshold growth rate and relatively independent of the turgor value. This is despite the fact that the instantaneous growth rate correlates with the turgor during transient turgor changes and despite the fact that the main driving force for the growth is the turgor pressure, as embodied by the Lockhart equation. Thus our model predicts that the average growth rate will be insensitive to the turgor value if the period of the oscillations is short, but that it is sensitive when the period of oscillations is long. Evidence for both situations, independence and dependence of the average growth rate on turgor, is available (Figs. 5D,E; [23]).

### Modulation of the transmembrane calcium flux

**Figure 7:**
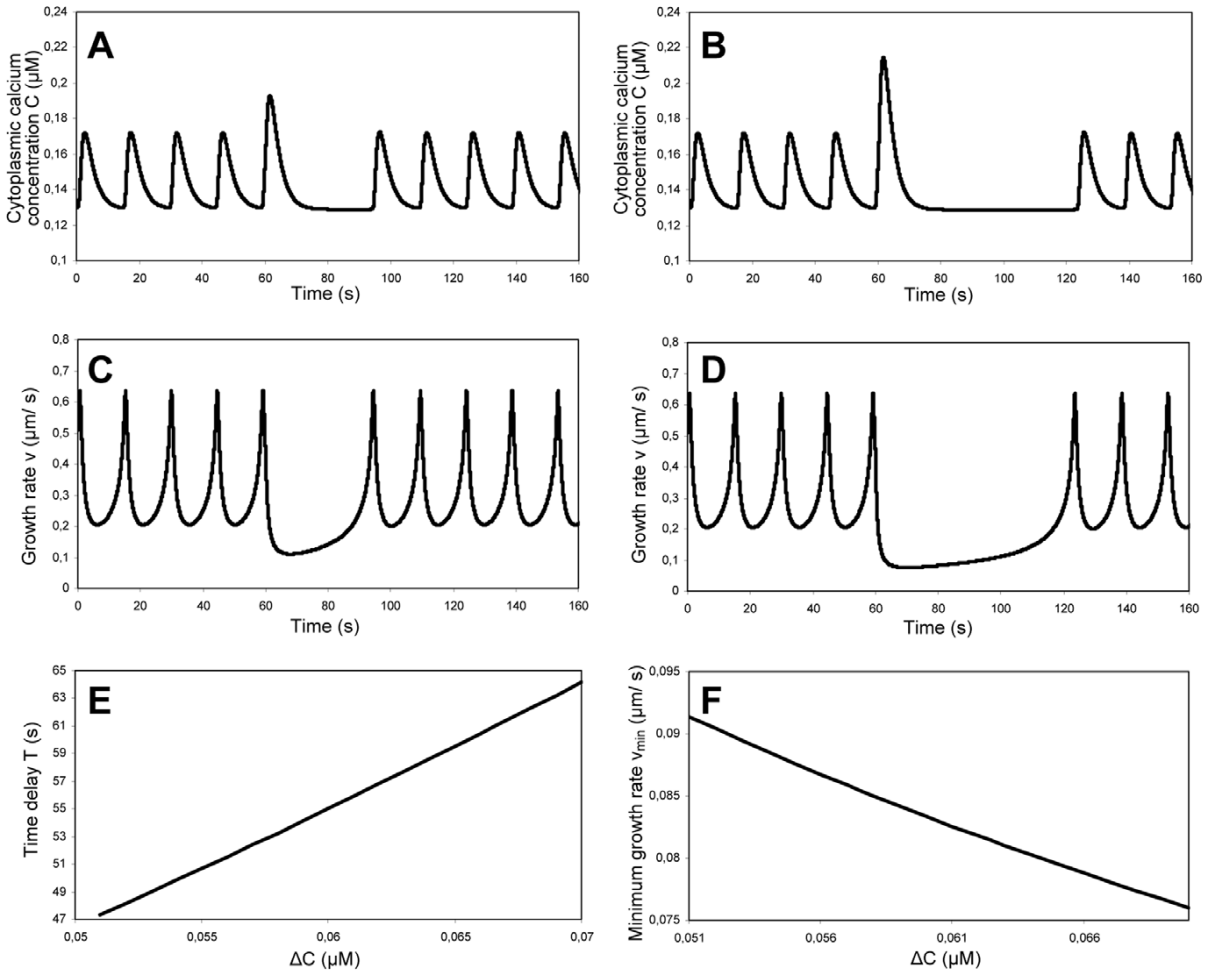
Simulation of the response of an oscillating pollen tube to a transient increase in calcium influx. Apical cytoplasmic calcium concentration (A, B) and growth rate (C, D) for an influx of 53 mM (A, C) and 70 mM (B, D) at t = 60 s. (E) Time delay T between the transient calcium increase and the subsequent maximum in the cytoplasmic calcium concentration for various calcium increases ΔC. (F) Minimum growth rate v_min_ for various calcium increases ΔC.

Our explanation for the absence of correlation between the turgor and the average growth rate is based on an upper bound on the growth rate set by the exocytosis mechanism. In this section, we investigate how calcium concentration affects exocytosis and how calcium uptake can increase the period of oscillation. To study the effect of an increased uptake of calcium ions into the cytoplasm, we simulate a raise in the cytosolic calcium concentration at a precise moment. The uptake transient, modeled by the equation
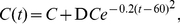
(17)


is maximal at 65 s (Figs. 7A, B). We observe that such an additional uptake delays the following concentration maximum by an interval T that is proportional to the amount ΔC (Fig. 7A, B, E). This can be explained by the response of the growth rate to the calcium uptake (Figs. 7C, D). The calcium ions induce an increased exocytosis activity and thus induce a thickening of the cell wall. This increase in exocytotic activity produces an immediate drop in the growth rate, since the growth rate is proportional to the cell wall stress which is inversely proportional to the cell wall thickness (eq. 3). This is consistent with experimental data demonstrating that photoactivation of caged calcium in the cytoplasm of growing pollen tubes causes a transient reduction in the growth rate [47], [48]. While the calcium triggered exocytosis can also be expected to cause an overall softening of the cell wall through the addition of new, highly methyl-esterified pectic polymers, our simulations suggest that the increase in thickness has the more immediate effect on the growth rate. The drop and the subsequent minimum in the growth rate depend on the amount of cell wall material added and are thus directly proportional to the amount of calcium taken up (Fig. 7F). The time necessary to reach the subsequent maximum depends directly on the minimal value, and thus on the calcium uptake.

### A framework for models of tube growth

Most of our simulations were carried out using a constant turgor pressure, implying our assumption that turgor pressure is mostly constant throughout tube growth and not responsible for the oscillations in the tube growth rate. However, we emphasize that the model presented here aims at a general formalism for the modeling of pollen tube growth, i.e. the identification of the different coupled variables that govern the growth and their feedback [4], [14], [16], rather than a definitive model for the tube growth. Indeed, recent experiments suggest that the pollen tube growth oscillation is not governed by a single “pacemaker” parameter that can oscillate independently [49]. Rather, the oscillation is believed to be an emergent property arising from positive and negative feedback loops, i.e. a network of coupled variables, interacting chemically and mechanically on different time scales, that oscillate with the same period but with different phase delays.

Since the publication of the first version of our model [34], two other mathematical models linking calcium dynamics and pollen tube growth behavior have been published [50], [51]. They are based on different feedback loops and explain different types of experimental data. Yan et al. [50] study the interactions between ROP1 GTPases, F-actin proliferation and calcium, and explain how drugs interfering with the functioning of the actin cytoskeleton, latrunculin B and jasplakinolide, disrupt the calcium oscillations. Liu et al. [51] model the interactions between different ionic currents, their gating variables and the trans-membrane potential. They show that these currents can sustain oscillations even in the absence of tube growth, a phenomenon that has been observed experimentally [52]. Here and in Kroeger et al. [34] we coupled the growth rate to the cell wall thickness and stress gated calcium channels, explaining the observed phase-lag between growth rate and apical cell wall thickness [53]. The main difference between these models is the mechanism that is assumed to be responsible for calcium entry at the tube apex, i.e. through voltage-gated channels [51], stress-activated channels [34] or the action of the scaffolding protein RIC3 [50]. These mechanisms are not mutually exclusive and it is possible that all of them contribute to calcium entry and thus the oscillation in cytosolic calcium concentration. It is also possible that these mechanisms operate in concert to render pollen tube growth robust against external perturbations. However, more experimental work is clearly necessary to establish whether, or when, one of these mechanisms dominates calcium dynamics in the growing pollen tube.

**Figure 8:**
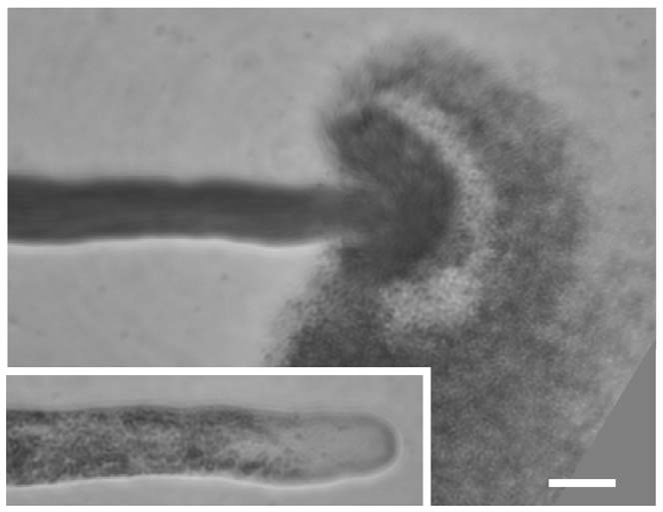
Pollen tube bursting at the apex. Brightfield micrograph of *Lilium* pollen tube 1 s after bursting at the apex. Cytoplasm is released quickly into the surrounding medium after failure of the cell wall to resist turgor. Inset shows intact tube immediately prior to bursting. Bar = 20 µm.

In addition to pressure, calcium concentration and rheological properties of the cell wall [15], [21], [26], [45], [50], we add the cell wall thickness as a governing variable of the growth rate. In addition to the recent observation of its oscillation during tube growth [53], thickness provides a negative feedback mechanism that is necessary to prevent the tube from bursting. The relation between the growth rate and the extensibility of the cell wall material is a positive feedback: as the growth rate increases and exceeds some critical value that triggers calcium influx and exocytosis, additional soft material is included in the cell wall. Exocytosis decreases the viscosity of the cell wall, i.e. increases the extensibility, which would, in the absence of any negative feedback, increase the growth rate even more. The extensibility thus provides positive feedback to the growth rate and increases any fluctuation. The presence of negative feedback is necessary to stabilize the process and prevent an exponential increase and a bursting of the cell. If we inhibit the secretion (exocytosis) in our model by reducing a_2_ below 3.6×10^−5^ µm/s/µM, the cell wall thickness can decrease below zero, a condition equivalent to the bursting of the tube, a phenomenon that occurs frequently during experimentation and almost always happens at the tip of the cell (Fig. 8).

Our explanation for the lack of correlation between the turgor pressure and the average growth rate, a threshold mechanism that limits the increase in the average growth rate, is not restricted to our particular model for pollen tube growth. Alternate mechanisms for growth rate oscillations such as turgor changes [15] could also lead to a lack of correlation if they presented threshold dynamics. The present model shows how the pollen tube growth mechanism is robust and flexible at the same time, a characteristic shared with other biological systems that allows them to function despite variations in the parameters or the biochemical environment [54], [55], [56].

## Materials and Methods

### Pollen tube growth

Pollen was collected from fresh flowers, dehydrated in gelatin capsules on anhydrous calcium sulfate overnight and stored at −20°C. Pollen was rehydrated in humid atmosphere for 30 minutes before cultivation. The growth medium for *Lilium* contained 0.16 mM H_3_BO_3_, 0.13 mM Ca(NO_3_)_2_, 1 mM KNO_3_, 5 mM MES, 100 mg mL^−1^ sucrose, pH 5.5. Unless specified otherwise, the medium for *Nicotiana* and *Petunia* pollen was composed of 100µg mL^−1^ H_3_BO_3_, 300 µg mL^−1^ Ca(NO_3_)_2_ H_2_O, 100 µg mL^−1^ KNO_3_, 200 µg mL^−1^ MgSO_4_ 7H_2_O, 120 mg mL^−1^ sucrose (modified after [57]). For the mannitol data set, the medium contained 70 mg mL^−1^ sucrose and was complemented with 16 or 32 mg mL^−1^ mannitol to reach the same osmolarity as that of media containing a total of 100 or 130 mg mL^−1^ sucrose, respectively.

### Time lapse imaging

Time lapse imaging for growth rate measurements were carried out on pollen growing on the surface of a thin layer of agarose as described previously [58]. Altered sucrose and mannitol concentrations were administered replacing the liquid layer of medium with medium containing the substance in question. Quantitative analysis of the growth rate was carried out as described previously [58] or using image acquisition with a Roper fx cooled CCD camera and the tracking function of the ImagePro software (Media Cybernetics). Plots of the growth rate were smoothed with local regression using weighted linear least squares.

## Supporting Information

Text S1 **Analytical derivation, based on a separation of time scales, of** **equation 13** **representing a relationship between the pollen tube**'**s average growth rate and a constant turgor pressure.** This analysis, and the ensuing equation, allows to interpret the results of the numerical simulations shown in Figure 3A.
(DOC)
