# Supplementary material for: Regulator or Driving Force? The Role of Turgor Pressure in Oscillatory Plant Cell Growth
Source: PLoS One. 2011 Apr 25;6(4):e18549. doi: 10.1371/journal.pone.0018549 (PMC3081820; doi:10.1371/journal.pone.0018549)
Supplement: Text S1 — Analytical derivation, based on a separation of time scales, of equation 13 representing a relationship between the pollen tube's average growth rate and a constant turgor pressure. This analysis, and the ensuing equation, allows to interpret the results of the numerical simulations shown in Figure 3A. (DOC) [file pone.0018549.s001.doc]

# Text S1

### Slow-fast analysis

The following analysis is used to calculate the average growth rate and the period of oscillation of our model. It starts by assuming that the cell wall viscosity and the cytosolic calcium concentration vary fast with respect to the growth rate and the cell wall thickness. Separation of time scales is frequently used to simplify systems of non-linear equations [1,2]. During most of the oscillation cycle, the viscosity and the calcium concentration barely change. Only when the growth rate reaches its threshold value *v*_c_, which is accompanied by a high membrane stress and by the opening of the membrane channels, do the viscosity and calcium concentration change in a rapid manner. The dynamics of the viscosity and calcium concentration can thus be separated into two distinct regimes: a slow regime when *v* < *v*_c_ and a fast regime when v > v_c_ (see Figure 2 in the main part of the article). Since the slow regime lasts much longer than the fast regime, we can approximate the period of the growth rate oscillation by the time it stays in the slow regime. This time is equal to the time interval during which the thickness decreases from its maximal to its threshold value. We calculate this time interval by combining eqs. 2, 3 and 5 in the main part of the article, to obtain

$\frac{\partial h}{\partial t}=\frac{-3(r^{2}-r_{i}^{2})}{2r}\Phi(\frac{rP}{2h}-\sigma_{y})+R$. (18)

Here we assumed that the arc length S and the outer cell radius r are equal to the radius of curvature of the cell wall r. By assuming that r, Φ and R are constant during the slow regime, we can simplify this differential equation to

$\frac{\partial h}{\partial t}=\frac{-A}{h}+R^{'}$(19)

with the constants A and R’ given by

$A=\frac{3\Phi P(r^{2}-r_{i}^{2})}{4}$ (20)

and

$R^{'}=R+\frac{3(r^{2}-r_{i}^{2})\Phi\sigma_{y}}{2r}$. (21)

The differential equation 19 can easily be solved to yield

$t=\frac{1}{R^{2}}(\ln(Rh-A)+Rh-A)$ (22)

and the time interval T = t_2_ – t_1_ needed for the thickness to change from value h_1_ to h_2_

. (23)

Calculating from typical values of the parameters (Table 1 in the main part of the article), A is of the order of 2.8×10^-5^ μm^2^/s. Assuming that σ_y_ is of the order of 0.1 MPa, we obtain

MPa^-1^ min^-1^. (24)

We finally estimate the value of R’ at 0.03 μm/s. In order to obtain an expression for the average growth rate, we solve eq. (19) for h by replacing the logarithmic function by its Taylor expansion

$\ln(Rh-A)\cong\ln(-A)-\frac{Rh}{A}-\left(\frac{Rh}{A}\right)^{2}$. (25)

This yields

$h(t)=\sqrt{K-At}$(26)

and it is a good approximation of the complete model until h(t) reaches its threshold value (e.g. h_2_) at which point it is reset to its maximal value (e.g. h_1_) by exocytosis. The constant *K* stands for

$K=\frac{A^{2}}{R^{2}}(\ln(-A)-1)$. (27)

We can now calculate the average thickness over one cycle $T=t_{2}-t_{1}$ by evaluating

$\bar{h}=\frac{1}{T}\int_{t_{1}}^{t_{2}} h(t)dt$. (28)

This expression can be used to calculate an average growth rate. However, we choose to linearize eq. (26) in order to obtain a tractable expression of the average thickness which we can use to obtain an average growth rate. This linearization is analogous to those used to reduce physiologically relevant models of periodically firing neurons [3] to simple phenomenological models such as integrate-and-fire models [4]. By keeping the first two terms of the binomial expansion of eq. (26), the model comprised of equations 5, 7, 11 is reduced to

$h(t)=\sqrt{K}-\frac{At}{2\sqrt{K}}$, (29)

with the condition

$h\to h_{2}+\tau R=h_{1}$ $if$ $h=h_{2}$. (30)

Equations 29 and 30 describe a tube whose cell wall gets thinner as the tube expands. The viscosity η and the secretion rate R are treated as constants during the integration of equation 29. Once it reaches the minimal or threshold value h_2_, the massive exocytosis resets the cell wall thickness to its maximal value h_1_. The time constant τ, of the order of a few seconds, is the short time interval needed to reset the cell wall thickness. A further assumption is that the growth rate is constant such that the cell wall thickness h decreases linearly with time. As can be seen from figure 2 in the main article, this assumption is reasonable for a large fraction of the growth cycle. If h decreases linearly from h_1_ to h_2_, its average value will simply be

$\bar{h}=\frac{h_{1}+h_{2}}{2}=h_{2}+\frac{\tau R}{2}$. (31)

Combining equations 2, 3 and 4 yields

, (32)

where we set σ_y_ to zero. The value of the minimal, threshold value of the cell wall thickness h_2_ is related to the critical value v_c_ at which the calcium channels become conductive. Furthermore, *v*_c_ is a constant input parameter of the model such that the minimal value h_2_ becomes the constant

$h_{2}=\frac{r^{2}\Phi P}{2v_{c}}$ . (33)

Replacing the expression into equation 31 yields

$\bar{h}=\frac{r^{2}\Phi P}{2v_{c}}+\frac{\tau R}{2}$. (34)

The average growth rate becomes

. (35)

Although it is based on many simplifications, this expression has the same shape as the numerical solution of the whole model (Fig 3A, solid line).

### References

1. Rinzel J (1985) Bursting oscillations in an excitable membrane model, in ordinary and partial differential equations. In: Sleeman BD, Jarvis RJ, editors. Lecture Notes in Mathematics. New York: Springer. pp. 304-316.

2. Izhikevich E (2000) Phase equations for relaxation oscillators. SIAM Journal of Applied Mathematics 60: 1789.

3. Hodgkin A, Huxley A (1952) A quantitative description of membrane current and its applications to conduction and excitation in nerve. Journal of Physiology 117: 500-544.

4. Gerstner W, Naud R (2009) How good are neuron models? Science 326: 379-380.
